# The effect of respiratory capacity for dose sparing in left-sided breast cancer irradiation with active breathing coordinator technique

**DOI:** 10.3389/fonc.2022.989220

**Published:** 2022-10-03

**Authors:** Hongtao Chen, Ying Piao, Dong Yang, Peipei Kuang, Zihuang Li, Guixiang Liao, Heli Zhong

**Affiliations:** Department of Radiation Oncology, Shenzhen People's Hospital, the Second Clinical Medical College, Jinan University, Shenzhen, China

**Keywords:** ABC, DIBH, radiotherapy, breast cancer, cardiac sparing

## Abstract

**Background and aim:**

A subsequent cardiac toxicity is deemed to be dose-dependent for left-sided breast cancer irradiation. This study aims to demonstrate the effect of respiratory capacity for dose sparing when the deep inspiration breath hold with Active Breathing Coordinator technique (ABC-DIBH) is used in left-sided breast cancer irradiation.

**Methods:**

74 left-sided breast cancer patients, who received whole breast or post-mastectomy chest wall radiotherapy with ABC-DIBH between 2020 and 2021 in our center, were retrospectively reviewed in this study. CT scans of free breath (FB) and ABC-DIBH were done for each patient, and two treatment plans with a prescription dose of 5000 cGy/25 Fr were designed separately. The dose to heart, left anterior descending artery (LAD) and lungs was compared between FB and ABC-DIBH. The correlation between individual parameters (dose to organs at risk (OARs) and minimum heart distance (MHD)) was analyzed, and the effect of respiratory capacity for dose sparing was assessed.

**Results:**

The plans with ABC-DIBH achieved lower Dmean for heart (34.80%, P < 0.01) and LAD (29.33%, P < 0.01) than those with FB. Regression analysis revealed that both Dmean and D2 of heart were negatively correlated with MHD in the plans with FB and ABC-DIBH, which decreased with the increase in MHD by 37.8 cGy and 309.9 cGy per 1mm, respectively. Besides, a lower Dmean of heart was related to a larger volume of ipsilateral lung in plans with FB. With the increase in volume of ipsilateral lung, the linear correlation was getting weaker and weaker until the volume of ipsilateral lung reached 1700 cc. Meanwhile, a negative linear correlation between Dmean of LAD and MHD in plans with FB and ABC-DIBH was observed, whose slope was 162.5 and 135.9 cGy/mm, respectively. Furthermore, when the respiratory capacity of ABC-DIBH reached 1L, and the relative ratio (ABC-DIBH/FB) reached 3.6, patients could obtain the benefit of dose sparing. The larger difference in respiratory capacity had no significant effect in the larger difference of MHD, Dmean of heart and Dmean of LAD between FB and ABC-DIBH.

**Conclusion:**

This study demonstrates the sufficiently good effect of ABC-DIBH when utilizing for cardiac sparing. It also reveals the correlations among individual parameters and the effect of respiratory capacity for dose sparing. This helps take optimal advantage of the ABC-DIBH technique and predict clinical benefits.

## Introduction

Adjuvant radiotherapy is an important part of curative-intent treatment for patients after breast conservation surgery or mastectomy (1). Adjuvant radiotherapy contributes to a favorable prognosis and reduces the risk of local-regional recurrence compared to surgery alone (2–4). However, toxicities associated with radiotherapy compromise the quality of life post-treatment (5). Particularly, due to the tangential fields, large amounts of radiation dose may locate in the anterior part of the heart, including the left anterior descending (LAD) artery, which is one of the structures closest to PTV. Incidental radiation dose to the left ventricle and LAD results in an increased risk of ischemic heart disease for left-sided breast cancer patients (6–9). Although there is no clear dose threshold for radiation-induced cardiac complications, the excess risk of ischemic heart disease increases linearly with the mean heart dose, which is evident within four years after radiotherapy and even continues for decades (7, 10). The study based on 2168 women who underwent radiotherapy for breast cancer by Darby et al. found that the rate of coronary events increased linearly with the Dmean of heart by 7.4% per Gy (7).

**Figure 1:**
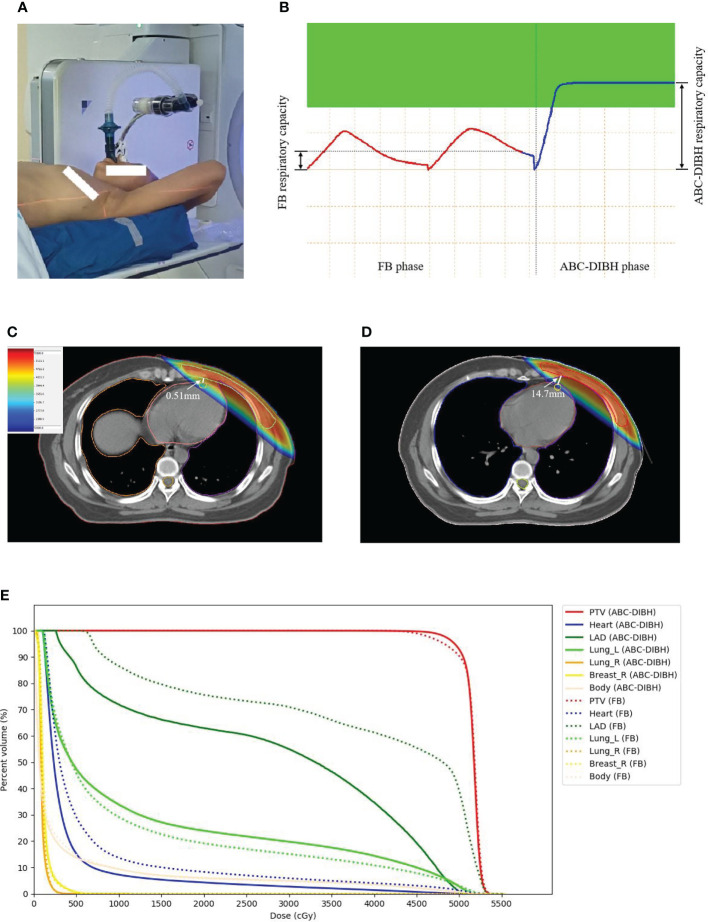
**(A)** ABC device applied in left-sided breast cancer radiotherapy. **(B)** FB and ABC-DIBH breathing curves. **(C)** Dose distribution on CT image in FB plan. **(D)** Dose distibution on CT image in ABC-DIBH plan. **(E)** DVH of structures in FB plan and ABC-DIBH plan.

Improvements in techniques of radiotherapy minimize the dose to heart and cardiac structures over the years, such as patient positioning methods (11), gating (12) and proton therapy (13). Respiratory management is regarded as another promising strategy applied in breast cancer radiotherapy (14–20). Accuracy of PTV dose delivery and protection of organs at risk (OARs) is adversely affected by respiratory motion. Deep inspiration breath hold with the Active Breathing Coordinator device (ABC-DIBH) can minimize breathing motion and consequently augments cardiac sparing in radiotherapy of breast cancer by increasing the distance between heart and PTV. In contrast to self-sustained breath hold (4.1% variability of lung volume), the ABC device was proved with better intra-session and inter-session reproducibility of respiratory capacity (1.8% and 3% variability of lung volume) on account of the function that induced breath hold automatically at a preset inhaled or exhaled air volume during a predetermined time (14, 15). Note that the ABC device utilizes the sensor to count the rotations of turbine impeller for a known respiratory capacity. Once the inhaled or exhaled air volume reaches the preset threshold, the balloon valve shuts off and stops airflow (**Figure 1A**).

In the ABC-DIBH feasibility study, Kunheri et al. demonstrated that the mean dose to the heart and LAD was reduced by 48.5% and 53.81% (ABC-DIBH VS. Free breath (FB)) (1). The study by Quirk et al. presented that the median heart dose and the median LAD dose in deep inspiration breath hold (DIBH) cohort are 10% lower than those of FB cohort (5). However, the samples of the two cohorts are different and without one-to-one correspondence. Eldredge-Hindy et al. showed that the median values of the relative and absolute reduction of mean heart dose were 62% and 1.7 Gy as use of the ABC-DIBH (21). The above studies have indicated that left-sided breast cancer patients benefited greatly from the ABC-DIBH technique, but there lacked consideration for individual parameters. More correlations among dose of OARs minimum heart distance (MHD), lung volume and inspiratory capacity are expected to reveal (22).

In this study, we have examined the individual parameters of dose and volume in left-sided breast cancer patients treated with adjuvant radiotherapy with FB or ABC-DIBH to demonstrate the advantages of respiratory management. Moreover, the novel objective was to characterize implicit correlations of individual parameters and assess the relationship between the dose of OARs and MHD. Despite the clear evidence of ABC-DIBH for reducing the dose to heart and LAD, the effect of respiratory capacity for dose sparing has remained unknown. This study quantified these correlations and effects that would help to take optimal advantage of the ABC-DIBH technique and predict clinical benefits.

## Materials and methods

74 left-sided breast cancer patients were retrospectively reviewed in this study who received whole breast (41 patients) or post-mastectomy chest wall (33 patients) radiotherapy with ABC-DIBH technique between 2020 and 2021 in our center. The median age was 43 (27-64). Patient characteristics are shown in **Table 1**. These retrospective data were deidentified and approved by the ethics committee of our institute. For this retrospective study, formal consent was not required. Eligible patients were required with no history of cardiac and lung diseases, no previous radiotherapy of the breast, and breath hold for 40 seconds at 80% of maximum deep inspiration. All of these patients underwent multiple simulations of thoracic breathing with the ABC device from Elekta before CT scan. The difference of position of mark point on the patient’s body among multiple deep inspiration breath holds was less than 2mm (5, 23, 24). All patients were scanned and treated in a supine position with arms above the head. CT scans of FB and ABC-DIBH with a slice thickness of 2.5 mm were acquired for per patient successively. On each CT scan, OARs and PTV were contoured by the same qualified physician as per the Radiation Therapy Oncology Group breast contouring guidelines. Both treatment plans were done by the same and qualified physicist in the Monaco planning system. Monte Carlo algorithm was adopted in all plans. The clinical treatment plans were prescribed according to the condition of individuals and the discretion of physicians, which was 5000 cGy/25 Fr, 4320 cGy/16 Fr or 4050 cGy/15 Fr. To ensure consistency, all plans adopted an experimental prescription dose of 5000 cGy/25 Fr in the final analysis. 95% of the prescribed dose covered at least 95% of the PTV volume while the dose to OARs was minimized as much as possible. These patients with whole breast radiotherapy also received sequential tumor bed boost delivered by electron ray or X ray, which depended on the physician’s consideration. Boost dose therefore was not included in the analysis.

**Table 1:** Patient characteristics.

Characteristic	Patients
Total patients	74
Age, median (range)	43 (27-64)
Stages	
IA	26
IB	1
IIA	17
IIB	16
IIIA	7
IIIB	1
IIIC	4
IVA	2
Surgery	73
Breast conserving surgery	40
Implant	5
Chemotherapy	52
ER (+)	53
PR (+)	51
HER-2 amplification	20

For each patient, delineated structures volumes (i.e., PTV, lungs, heart) were documented for both CT scans, and dose volume histograms (DVHs) were generated for these structures in both treatment plans. For PTV, conformal index (CI) and homogeneity index (HI) were calculated to assess the dose coverage. For heart, D2, Dmean, V30, V20, V10 and V5 were recorded. For LAD, Dmax, Dmean, V40, V30 and V20 were recorded. For ipsilateral lung, Dmean, V20 and V5 were recorded. The minimum heart distance (MHD), defined as the minimum vertical distance from the posterior edge of PTV to the heart border, was measured to detect the variation of location between heart and PTV in both plans. Moreover, the respiratory capacity with ABC-DIBH for consistent breath-holds during CT scan was recorded for each patient (**Figure 1B**). The waveform of respiratory capacity with FB was similar to a sine wave. CT reconstruction of FB was performed on all respiratory phases. The end expiration of FB was set as the origin. The amplitude of free breathing curve was considered as respiratory capacity with FB (**Figure 1B**).

SPSS 20.0 software was used for statistical analysis. The calculated data was expressed as mean and standard deviation. The paired t-test was adopted to analyze these statistic variables. P < 0.05 was considered as a statistically significant difference. Additionally, regression analysis was performed to search for correlations between these parameters. The Pearson test (r) and Spearman test (ρ) were adopted to assess the correlation between MHD and dose to heart, MHD and dose to LAD, volume of ipsilateral lung and dose to heart, respectively.

## Results

**Figure 2:**
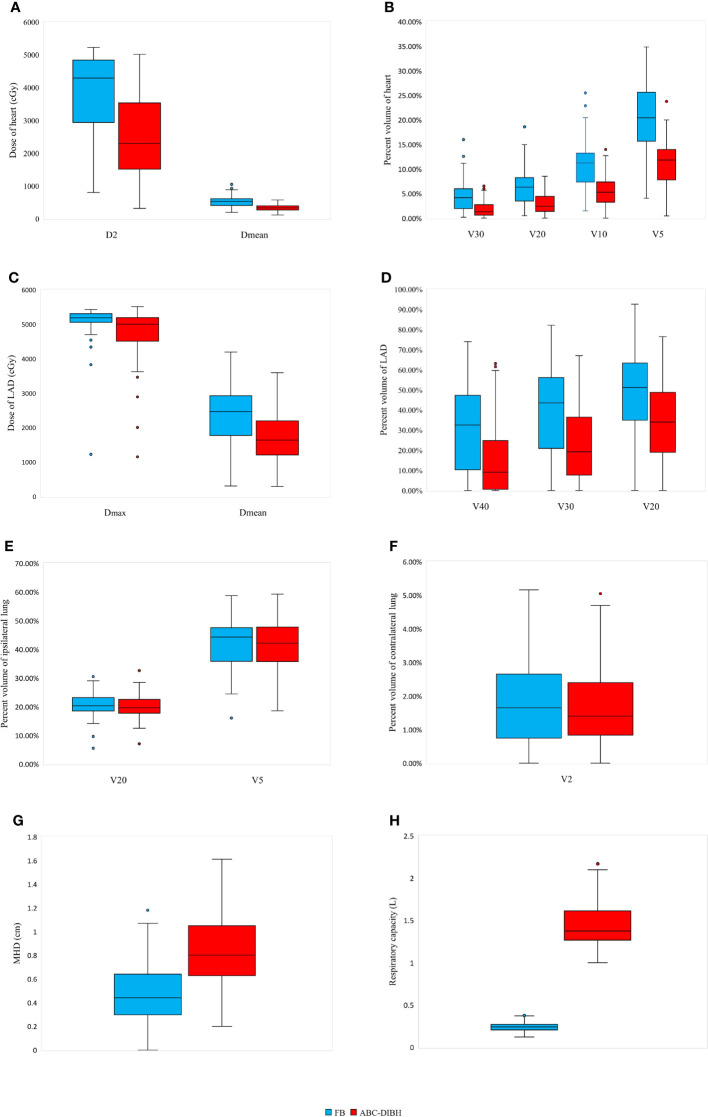
Distribution of parameters of heart (**A**: D2 and Dmean; **B**: V30, V20, V10 and V5), LAD (**C**: Dmax and Dmean; **D**: V40, V30 and V20), ipsilateral lung (**E**: V20 and V5), contralateral lung (**F**: V2), MHD **(G)** and respiratory capacity **(H)** with FB and ABC-DIBH.

All the 74 patients’ data of 148 CT scans and 148 treatment plans were analyzed. Patients’ variability was noted in volume and dose parameters of PTV and OARs. As shown in **Table 2**, the PTV volume was comparable and without significant difference between the FB and ABC-DIBH plans. CT scans with ABC-DIBH showed significantly larger ipsilateral lung and contralateral lung than those with FB, where the mean increase was 53.83% and 46.41% under P<0.01, respectively. Although the ABC-DIBH increased the intrathoracic pressure and thus enlarged the distance between the heart and PTV (an increase of 82.61% for MHD, distribution shown in **Figure 2G**), no significant variation in heart volume was observed. Moreover, in comparison with FB, the average respiratory capacity of ABC-DIBH increased by 495.83% (P < 0.01, **Figure 2H**).

**Table 2:** Volumes and MHD between FB and ABC-DIBH plans.

	FB	ABC-DIBH	P value
PTV (cc)	631.16 ± 221.32	636.97 ± 227.55	0.18
Ipsilateral lung (cc)	1175.95 ± 245.96	1808.96 ± 286.77	0.00
Contralateral lung (cc)	1435.70 ± 240.91	2102.03 ± 299.68	0.00
Heart (cc)	537.38 ± 99.75	529.94 ± 86.38	0.23
MHD (cm)	0.46 ± 0.29	0.84 ± 0.32	0.00
Respiratory capacity (L)	0.24 ± 0.05	1.43 ± 0.24	0.00

FB, free breath; ABC-DIBH, deep inspiration breath hold with Active Breathing Coordinator technique; MHD, minimum heart distance.

While ensuring prescribed dose coverage of the PTV (FB, CI=0.70, HI=1.09; ABC-DIBH, CI=0.71, HI=1.09), exposure to OARs was decreased as much as possible. As shown in **Table 3**, the plans with ABC-DIBH achieved distinctly lower D2, Dmean, V30, V20, V10 and V5 of heart than the plans with FB, where the relative reductions were 32.72%, 34.80%, 58.78%, 52.74%, 48.30% and 44.31% under P<0.01, respectively. The distribution of these parameter values was presented in **Figures 2A, B**. Meanwhile, it relatively reduced the Dmax, Dmean, V40, V30 and V20 of LAD by 8.47%, 29.33%, 53.00%, 42.69% and 32.31%, P<0.01, whose distribution was observed in **Figures 2C, D**. In addition, the plans with ABC-DIBH outperform FB on V20 reduction of ipsilateral lung (3.12%, P<0.05, **Figure 2E**) and Dmean reduction of contralateral lung (4.82%, P<0.01). Whereas, there is no significant reduction for the Dmean and V5 of ipsilateral lung (7.86%, 0.47%) and V2 of contralateral lung (7.18%, **Figure 2F**) in both plans.

**Table 3:** Dosimetric parameters of PTV and OARs between FB and ABC-DIBH plans.

Parameters		FB	ABC-DIBH	P value
PTV	CI	0.70 ± 0.06	0.71 ± 0.05	0.00
	HI	1.09 ± 0.02	1.09 ± 0.03	0.33
Heart	D_2_ (cGy)	3774.77 ± 1297.68	2539.69 ± 1295.97	0.00
	Dmean (cGy)	520.83 ± 181.42	339.56 ± 100.57	0.00
	V30 (%)	4.44 ± 3.24	1.83 ± 1.65	0.00
	V20 (%)	6.20 ± 3.79	2.93 ± 2.12	0.00
	V10 (%)	10.60 ± 4.89	5.48 ± 3.04	0.00
	V5 (%)	19.93 ± 7.97	11.10 ± 4.36	0.00
LAD	Dmax (cGy)	5145.65 ± 259.94	4709.84 ± 767.23	0.00
	Dmean (cGy)	2498.46 ± 828.79	1765.67 ± 740.22	0.00
	V40 (%)	33.17 ± 21.44	15.59 ± 16.76	0.00
	V30 (%)	42.68 ± 20.26	24.46 ± 19.04	0.00
	V20 (%)	51.28 ± 18.16	34.71 ± 19.17	0.00
Ipsilateral lung	Dmean (cGy)	1219.70 ± 500.35	1123.81 ± 204.36	0.08
	V20 (%)	20.54 ± 3.92	19.90 ± 4.07	0.04
	V5 (%)	42.11 ± 8.12	41.91 ± 8.41	0.79
Contralateral lung	Dmean (cGy)	83.62 ± 9.14	79.59 ± 9.78	0.00
	V2 (%)	1.81 ± 1.28	1.68 ± 1.19	0.29
Contralateral breast	Dmean (cGy)	111.18 ± 16.21	112.78 ± 17.34	0.13
	V5 (%)	0.24 ± 0.52	0.33 ± 0.60	0.08

CI, conformal index, CI=(V_P, ref_/V_P_) * (V_P, ref_/V_ref_), (V_P, ref_, the volume of PTV covered by the prescription dose, V_P_, the volume of PTV, V_ref_, the volume covered by the prescription dose); HI, homogeneity index, HI=D5/D95, (D5, the dose at 5% volume of PTV, D95, the dose at 95% volume of PTV); LAD, left anterior descending artery; Dmax, maximum dose; D2, the dose at 2% volume; Dmean, mean dose; Vx, the percent of volume covered by dose of x Gy.

**Figure 3:**
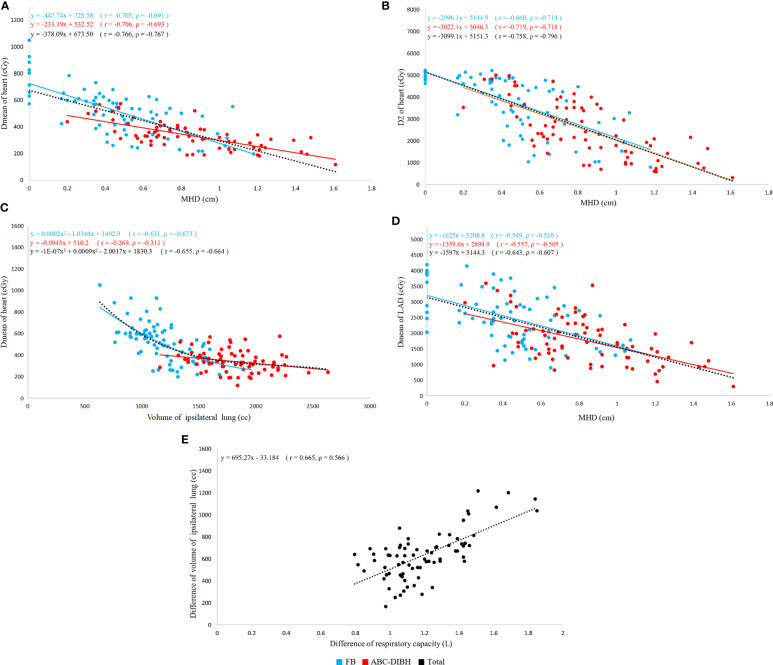
Correlation of individual parameters (r, Pearson’s rank correlation coefficient; ρ, Spearman’s rank correlation coefficient). **(A)** Correlation between Dmean of heart and MHD with FB and ABC-DIBH. **(B)** Correlation between D2 of heart and MHD with two modes. **(C)** Correlation between Dmean of heart and volume of ipsilateral lung with two modes. **(D)** Correlation between Dmean of LAD and MHD with two modes. **(E)** Correlation between difference of volume of ipsilateral lung and difference of respiratory capacity with two modes.

Regression analysis demonstrated that both Dmean and D2 of heart were negatively correlated with MHD in plans with FB and ABC-DIBH respectively, as shown in **Figures 3A, B**. In general, Dmean and D2 of heart decreased with the increase of MHD by 37.8 cGy and 309.9 cGy per 1mm, respectively. Meanwhile, a lower Dmean of heart was related to a larger volume of ipsilateral lung in plans with FB (r=-0.631, ρ=-0.673, **Figure 3C**). With the increase in volume of ipsilateral lung, this correlation was getting weaker and weaker until the volume of ipsilateral lung reached 1700 cc. Therefore, this correlation was nonsignificant in plans with DIBH (r=-0.269, ρ=-0.311). Furthermore, the analysis revealed a negative linear correlation between Dmean of LAD and MHD in plans with FB (r=-0.549, ρ=-0.510, **Figure 3D**) and ABC-DIBH (r=-0.557, ρ=-0.505, **Figure 3D**), whose slope was 162.5 and 135.9 cGy/mm, respectively.

**Figure 4:**
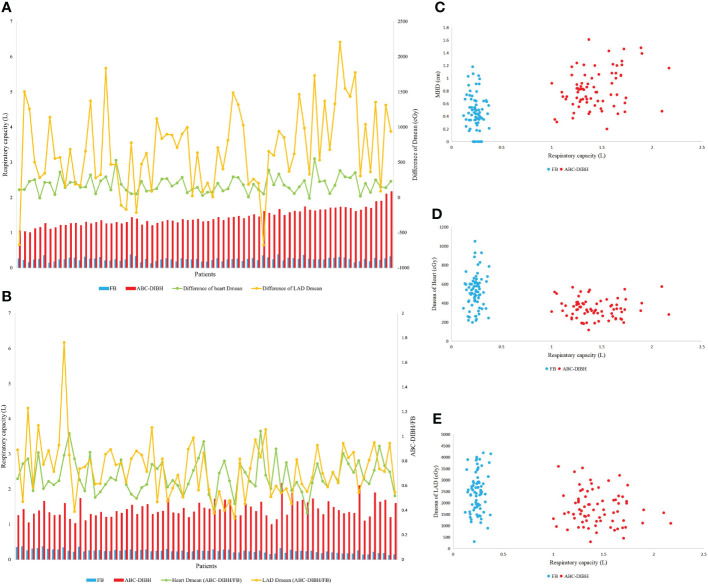
**(A)** Respiratory capacity, differece of Dmean of heart and LAD of per patient between FB and ABC-DIBH (in order of absolute difference of respiratory capacity). **(B)** Respiratory capacity, ratio of Dmean of heart and LAD of per patient between FB and ABC-DIBH (ABC-DIBH/FB, in order of ratio of respiratory capacity). **(C)** Distribution of MHD against respiratory capacity between FB and ABC-DIBH. **(D)** Distribution of Dmean of heart against respiratory capacity between FB and ABC-DIBH. **(E)** Distribution of Dmean of LAD against respiratory capacity between FB and ABC-DIBH.

In terms of respiratory capacity with FB and ABC-DIBH, a positive correlation between the difference of respiratory capacity and difference of ipsilateral lung volume was found (r= 0.665, ρ=0.556, **Figure 3E**). However, as revealed by **Figures 4A, B**, with the absolute difference and relative ratio of respiratory capacity between ABC-DIBH and FB increased, there lacked correlation for the difference of Dmean of heart. The same was true for the differences of Dmean of LAD. There was an apparent separation for MHD, Dmean of heart and Dmean of LAD plotted against respiratory capacity between the two groups (**Figures 4C-E**). The ABC-DIBH group had a larger MHD and lower overall Dmean of heart and LAD than the FB group without a distribution pattern. Hence, the larger difference of respiratory capacity between ABC-DIBH and FB had no significant effect on the larger difference of MHD, Dmean of heart and Dmean of LAD. In this study, when the respiratory capacity of ABC-DIBH reached 1L and the relative ratio (ABC-DIBH/FB) reached 3.6, patients could obtain the benefit of dose sparing from the ABC-DIBH.

## Conclusion and discussion

Radiation-induced toxicities are a severe concern for left-sided breast cancer patients, which not only discount the quality of life but also increase the likelihood of mortality due to the cardiac impairment and failure. Although the subsequent risk is uncertain, the excess events of ischemic heart disease increase linearly with the mean dose of heart. Respiratory motion management of ABC-DIBH is therefore an appropriate method applied in treatment delivery, which can decrease the heart and LAD exposure to radiation dramatically.

The results from our study not only demonstrate the encouraging effect that ABC-DIBH is utilized for the reduction of cardiac dose but also reveal the correlation among individual parameters in left-sided breast cancer patients treated with adjuvant radiotherapy. Similar to the results of our study, Azam Eskandari et al. in their study of 17 left-sided breast cancer patients had performed dosimetric comparison for FB and DIBH plans. They demonstrated that the DIBH plans achieved lower the Dmean of heart with respect to those with FB (3.83 Gy VS. 5.79 Gy) (25). C. S. Chang et al. in their review of 21 left-sided breast cancer patients acknowledged that the mean heart dose and mean LAD dose of DIBH plans was reduced by 41% and 42% separately. It also revealed that Dmean of heart and LAD were negatively correlated with the volume of ipsilateral lung. They suggested the difference of lung volume between the two groups could be adopted to screen patients for DIBH (22). Diana Lee et al. in their analysis of 47 patients treated with FB and 41 patients treated with ABC-DIBH had shown an inverse relationship between Dmean of heart and volume of left lung. Likewise, Dmean of LAD decreased with increasing volume of left lung for all patients (26). We can conclude that DIBH is a practical way for cardiac sparing in radiotherapy of left-sided breast cancer. ABC is employed for this purpose.

While for the reduction of dose to lungs by DIBH, there seems to be controversy. C. S. Chang et al. held that the DIBH had no significant reduction of V5, V20 and Dmean of ipsilateral lung and whole lung (22). Azam Eskandari et al. and Diana Lee et al. considered that DIBH would increase Dmean and V20 of ipsilateral lung despite the fact that it lacking of statistical significance (25, 26). However, Harriet et al. presented statistically significant reductions in the dose of ipsilateral lung and whole lung with ABC-DIBH (21). In our work, the plans with ABC-DIBH outperform FB on V20 reduction of ipsilateral lung and Dmean reduction of contralateral lung with statistical significance. Whereas, there is nonsignificant reduction for the Dmean and V5 of ipsilateral lung and V2 of contralateral lung in both plans. This is mainly because that while the volume of whole lungs becomes larger after inhalation, the part within or near the radiation fields also becomes larger without correspondence (**Figures 1C, D**) as the lower lobes of lung exhibit a higher degree of airflow exchange than other lobes (27, 28). The volume of exposure to radiation gets complicated (**Figure 1E**). In terms of dose to lung, not all patients can receive the significant benefit from the DIBH on account of some outliers. A larger number of samples are needed for this purpose in further investigations. Differently, Song et al. held that it is wrong as the larger absolute lung volume of exposure to radiation with DIBH results in an increased risk of radiation-induced lung toxicity (27). The density of functional units such as alveoli and bronchioles should be considered, which is negatively correlated with the volume of lung. Radiation damage to these functional units is more likely to lead to functional impairment (28). The effects may even be evaluated on a molecular level with the help of micronucleus testing (29).

We have found that MHD is a crucial factor which determines the cardiac dose. Dose to heart and LAD is negatively correlated with MHD in plans with FB and ABC-DIBH, respectively. The expansion of ipsilateral lung with DIBH pushes the heart away from the radiation field and broadens the MHD, which decreases cardiac dose by leaps and bounds. Nevertheless, it is patient-specific for the difference of lung volume between FB and ABC-DIBH, which doesn’t determine the difference of MHD and therefore doesn’t determine the difference of cardiac dose between the two modes. Similarly, although the difference of respiratory capacity is associated with the difference of lung volume between FB and ABC-DIBH, it is not associated with the difference of MHD, and thus was not associated with the difference of cardiac dose. In brief, deep inspiratory can increase lung volume and MHD, thereby reducing the cardiac dose. Whereas, it does not mean that more respiratory capacity decreases more cardiac dose. Our data demonstrate that patients can obtain the desired benefit when the respiratory capacity of ABC-DIBH reaches 1L and the relative ratio (ABC-DIBH/FB) reaches 3.6. It needs to be cautious that the threshold of inspiration volume is set too close to the maximum breath-hold. Comfort is the other factor that has to be considered. Angela et al. found through paper questionnaires that more than half of patients felt moderately to highly nervous and starved for air when using the ABC-DIBH technique (30). Discomfort may lead to rapid breaths and chaotic breath-holds (15). Therefore, an appropriate threshold is significant to keep the stable breath-hold with comfort when the respiratory capacity of ABC-DIBH reaches 1L, and the relative ratio (ABC-DIBH/FB) reaches 3.6.

In addition, the variation of airflow rate in a sense affects respiratory capacity measured by the ABC device. The work by Soyoung Lee et al. with 12 patients received ABC breath-hold treatment indicated a positive correlation that the recorded respiratory capacity increased as the airflow rate increased on inhalation mode. They measured air volume with a specific syringe at several airflow rates and confirmed the accuracy within 5% tolerance. In terms of respiratory capacity of patients, the maximum difference with respect to the reference volume of conventional radiotherapy and SBRT was 1.0 L and 0.16 L, with airflow rates of 0.77 L/s and 0.29 L/s range, respectively (15). The wide range of airflow rates of patients affects the actual measured inspiratory results, and thus the impact of this on our statistics cannot be ignored. It affects the repeatability in breath-hold volume during patients’ treatment fractions. Additionally, although ABC can monitor the breathing curve in real time, it cannot display the intra-fractional and inter-fractional position variation. If a patient has false breath-holding, such as leaking air from the corner of mouth or breathing through the nose, it is difficult to judge from the breath curve.

In conclusion, this study has demonstrated the significant impact on cardiac sparing from radiation on account of variation in lung volume or expansion, as well as suggests the effect of respiratory capacity in left-sided breast cancer irradiation with ABC-DIBH compared with FB. MHD plays a significant role which determines the cardiac dose. The larger difference of respiratory capacity has no significant effect on the larger difference of MHD, Dmean of heart and Dmean of LAD between ABC-DIBH and FB. When the respiratory capacity of ABC-DIBH reached 1L and the relative ratio (ABC-DIBH/FB) reached 3.6, patients could obtain the benefit of dose spaing. Further investigation of the effect of respiratory capacity considering the intra-fractional and inter-fractional variation will be implemented.

## Data availability statement

The original contributions presented in the study are included in the article/**Supplementary Material**. Further inquiries can be directed to the corresponding authors.

## Ethics statement

The studies involving human participants were reviewed and approved by The ethics committee of Shenzhen People’s hospital. Written informed consent for participation was not required for this study in accordance with the national legislation and the institutional requirements.

## Author contributions

HTC recorded and analyzed the data, and wrote the manuscript. YP, DY, PPK, ZHL and GXL helped with the statistical analysis. YP and HLZ revised the manuscript. All authors contributed to the study and approved the submitted manuscript.

## Conflict of interest

The authors declare that the research was conducted in the absence of any commercial or financial relationships that could be construed as a potential conflict of interest.

## Publisher’s note

All claims expressed in this article are solely those of the authors and do not necessarily represent those of their affiliated organizations, or those of the publisher, the editors and the reviewers. Any product that may be evaluated in this article, or claim that may be made by its manufacturer, is not guaranteed or endorsed by the publisher.
